# Clinicopathological and prognostic significance of GPX2 protein expression in esophageal squamous cell carcinoma

**DOI:** 10.1186/s12885-016-2462-3

**Published:** 2016-07-07

**Authors:** Zhijin Lei, Dongping Tian, Chong Zhang, Shukun Zhao, Min Su

**Affiliations:** Department of Pathology and Institute of Clinical Pathology, Shantou University Medical College, Shantou, Guangdong People’s Republic of China; Forensic Identification Center of Shantou University, Shantou University Medical College, Shantou, Guangdong People’s Republic of China

**Keywords:** ESCC, GPX2, Overexpression, Clinicopathological parameters, Prognosis

## Abstract

**Background:**

Chaoshan region, a littoral area of Guangdong province in southern China, has a high incidence of esophageal squamous cell carcinoma (ESCC). At present, the prognosis of ESCC is still very poor, therefore, there is urgent need to seek valuable molecular biomarker for prognostic evaluation to guide clinical treatment. GPX2, a selenoprotein, was exclusively expressed in gastrointestinal tract and has an anti-oxidative damage and anti-tumour effect in the progress of tumourigenesis.

**Methods:**

We collected 161 ESCC patients samples, among which 83 patients were followed up. We employed immunochemistry analysis, western blotting and quantitative real-time PCR for measuring the expression of GPX2 within ESCC samples. We analysed the relationship between the expression of GPX2 and clinicopathological parameters of 161 patients with ESCC by Chi-square or Fisher’s exact test. The survival analysis of GPX2 expression within ESCC tissues was evaluated by the Kaplan-Meier method and Cox-regression.

**Results:**

A significant higher expression level of GPX2 was detected in tumour tissues compared to that in non-tumour tissues (*P* < 0.001). Moreover, GPX2 expression has statistically significant difference in the tumour histological grade of ESCC (*P* < 0.001), while there was no statistically significant difference in age, sex, tumour size, tumour location, gross morphology and clinical TNM stages (*P* > 0.05). Meanwhile, the expression of GPX2 protein was obviously down-regulated within poorly differentiated ESCC. Last, survival analysis revealed that tumour histological grade and clinical TNM stages, both of the clinical pathological parameters of ESCC, were associated with the prognosis of patients with ESCC (respectively, *P* = 0.009, HR (95 % CI) = 1.885 (1.212 ~ 2.932); *P* = 0.007, HR (95 % CI) = 2.046 (1.318 ~ 3.177)). More importantly, loss expression of GPX2 protein predicted poor prognosis in patients with ESCC *(P* < 0.001, HR (95 % CI) = 5.700 (2.337 ~ 13.907)).

**Conclusions:**

Collectively, these results suggested that the expression of GPX2 was significantly up-regulated within ESCC tumour tissues. GPX2 might be an important predictor for the prognosis of ESCC and a potential target for intervention and treatment of ESCC.

**Electronic supplementary material:**

The online version of this article (doi:10.1186/s12885-016-2462-3) contains supplementary material, which is available to authorized users.

## Background

Esophageal carcinoma (EC) is one of the most common digestive tract malignancies worldwide, and respectively ranks 8^th^ and 6^th^ in terms of cancer incidence and mortality rate [1]. The Chaoshan area of Guangdong province in China is a high incidence district of EC, where the main histologic type of EC is esophageal squamous cell carcinoma (ESCC). For most patients with EC, the first time going to a doctor is usually started after dysphagia, which means most patients are diagnosed with EC in the mid-late stage of the disease. Unfortunately, the 5-year survival rate of patients with EC ranges from 6 to 50 %, but generally less than 30 % [2]. Thus, the early detection of tumour prognostic factors is essential to patients with EC.

Glutathione peroxidase 2 (GPX2), also known as the glutathione peroxidase (GI-GPX), belongs to the antioxidant enzyme glutathione peroxidase family. The antioxidant enzyme family has eight known glutathione peroxidases (GPX1-8) in human [3]. Interestingly, the antioxidant enzyme family obviously exhibits a tissue-specific expression [4]. GPX2 is exclusively expressed in gastrointestinal tract, but in human, GPX2 is also expressed in liver [5], and has been suggested to protect against oxidative damage from food [6]. At present, the overexpression of GPX2 protein is detected in neoplastic transformation of squamous epithelia cells [7], Barrett’s esophagus [8], lung cancer [9], breast cancer [10], colorectal cancer [11, 12], hepatocellular carcinoma [13] and castration-resistant prostate cancer [14]. Intriguingly, GPX2 may be a candidate as a prognostic marker for castration-resistant prostate cancer [14]. However, the expression of GPX2 within ESCC is rarely reported, and the relationship between GPX2 expression and ESCC prognosis is still unclear. In this study, we found that the expression of GPX2 protein was significantly up-regulated within ESCC tumour tissues compared with non-tumour tissues. More importantly, the expression of GPX2 protein might be correlated with the prognosis of patients with ESCC.

## Methods

### Patients and tissue specimen

**Fig. 1:**
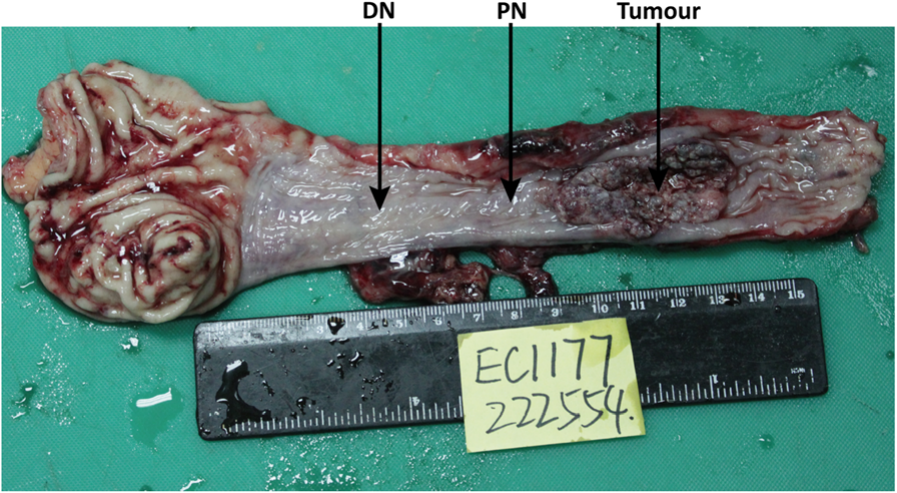
Schematic illustration of tissue sample collection. Samples without follow-up in this study were collected accordingly: tumour tissue (inside tumour), tumour-proximal non-malignant tissue (PN, within 2 cm from tumour) and distant non-malignant tissue (DN, over 5 cm away from tumour)

In this study, all tissue samples were collected from the Pathology Department of Shantou University Medical College, and their clinical pathological features and diagnosis were verified by two pathologists. Patients who were diagnosed with ESCC and with no radio- or chemo- preoperative therapy were enrolled in this study. We collected samples from 78 patients with ESCC in the year 2012 and separately sampled three sorts of these samples: tumour tissue (inside tumour), tumour-proximal non-malignant tissue (PN, within 2 cm from tumour) and distant non-malignant tissue (DN, over 5 cm away from tumour) (Fig. 1). According to the above standards, there were 60 patients with ESCC who has triple samples, namely tumour tissue, PN tissue and DN tissue. These tissue samples were in duplicate stored in −80°C freezer and embedded by paraffin. We also collected paraffin embedded samples from 83 patients with ESCC in the second half year of 2002 and followed up these patients or their families from July, 2002 to May, 2008. These samples with follow-up were only tumour tissues without PN and DN. On the basis of the 2010 WHO classification of tumours of digestive system [15], ESCC was divided into high differentiated ESCC (ESCCI), moderately differentiated ESCC (ESCCII) and poorly differentiated ESCC (ESCCIII). According to the 7^th^ editions of the Union for International Cancer Control-American Joint Committee on cancer (UICC-AJCC) TNM staging system [16], patients with ESCC was grouped into stages I, II, III and IV. This study was approved by the ethical board of Shantou University Medical College.

### Immunohistochemical analysis

Immunohistochemical staining was performed using the Envision Labelled Peroxidase System (Dako, carpinteria, CA). For immunohistochemical analysis, deparaffinised sections of the tissues were incubated with 1:100 diluted Pierce anti-GPX2 (Rabbit polyclonal antibody, PA5-27150, Thermo Fisher Scientific, Taiwan). The sections were allowed to develop in 3,3-diaminobenzidine (DAB). Negative [PBS instead of primary antibody or isotype-matched non-specific IgG (normal rabbit IgG, A7016, Beyotime, China) (Additional file 1: Figure S1)] and positive (gastric carcinoma) controls were used to confirm the specificity of the primary antibodies. After mounting, sections were scanned by Aperio ePathology AT2 (Leica Biosystems, Germany) and images were captured using a Aperio ImageScope (Version 12.1, Leica Biosystems, Germany) at 40× and 200× magnification. The quantitation of GPX2 cytoplasmic immunoreactivity was determinated by the product of the dyeing intensity and the expression rate. The dyeing intensity was divided into four degrees including 0, 1, 2 and 3. When the product was more than 0.5, the expression of GPX2 protein within ESCC tissues was defined as GPX2-positive expression (GPX2+); when less than 0.5, it was defined as GPX2-negative expression (GPX2-).

### RNA preparation and quantitative RT-PCR

The freshly tumour, PN and DN tissue samples from 7 patients with ESCC were stored in −80°C freezer. After thawing, total RNA was extracted from the freshly tissue samples using an RNeasy MiNi Kit (TianGen, Beijing, China). Reverse transcription was performed using a PrimeScript RT Master Mix Perfect Real Time 100 Reactions Kit (TaKaRa, Dalian, China). For measurement of GPX2 mRNA level, RT-PCR was performed with SYBR Premix EX TaqTM II (TaKaRa, Dalian, China). Primer sequences were 5’ - TGCAACCAATTTGGACATCAG - 3’ and 5’- AGACAGGATGCTCGTTCTGC-3’ for human GPX2; 5’-CAGCCTCAAGATCATCAGCA - 3’ and 5’ - ATGATGTTCTGGAGAGCCCC - 3’ for human glyceraldehyde-3-phosphate dehydrogenase (GAPDH). The following experiments were performed in triplicate in a 7300 Real-Time PCR System (ABI, USA). Analysis of relative GPX2 gene expression data was using real-time quantitative PCR and the the 2^−ΔΔCT^ Method [17].

### Western blotting analysis

The freshly tumour, PN and DN tissue samples from 6 patients with ESCC and freshly tumour tissue samples from other 6 patients with ESCC were lysed in RIPA buffer in the presence of 1 × protease inhibitor cocktail (Sigma-Aldrich, USA). 50 μg of total protein was resolved on 10 % sodium dodecyl sulfate-polyacrylamide gels and transferred onto PVDF membranes (0.45 μm, Amersham Pharmacia, USA). The expression level of GPX2 was assessed by Pierce anti-GPX2 (Rabbit polyclonal antibody, 24 kDa, 1:1000, PA5-27150, Thermo Fisher Scientific, Taiwan). Beta actin (β-actin) expression was evaluated to confirm equal amounts of protein loading using a mouse monoclonal anti-beta-actin antibody (42 kDa, 1:10000, Mab1445, Sigma-Aldrich, USA). After incubated with Goat anti-rabbit or mouse antibody (1:10000, 680RD, LI-COR biosciences, Germany), identification of the bands was scanned by Odyssey infrared imaging system (LI-COR biosciences, Germany). The outcome images were exported by Odyssey application software (Version 3.0, LI-COR biosciences, Germany).

### Statistical analysis

All data was analysed with SPSS statistics software (Version 19.0, Chicago, IL, USA). Relationships between GPX2 expression and ESCC clinicopathological features were studied using the Chi-square test or Fisher’s exact test. The GPX2 expression of tumor tissues compared with that of non-tumour tissues including PN and DN tissues was studied by the independent-samples test. Survival time was calculated from the date of surgery to the date of death or the last follow-up time. The correlation of different survival time with ESSC characteristics, clinical features and GPX2 were evaluated by using the Kaplan-Meier method. The log-rank test was used to analyse survival differences. The hazard ratio (HR) and 95 % confidence interval (CI) were calculated by univariate or multivariate Cox regression analysis. In order to identify the predictors of ESCC outcome, we used Cox stepwise regression for calculation with a significance level of *P* < 0.05 for entering and *P* > 0.10 for removing the respective explanatory variables. A *P* value of less than 0.05 was considered as statistically significant difference.

## Results

### GPX2 protein was overexpressed within ESCC tumour tissues

**Fig. 2:**
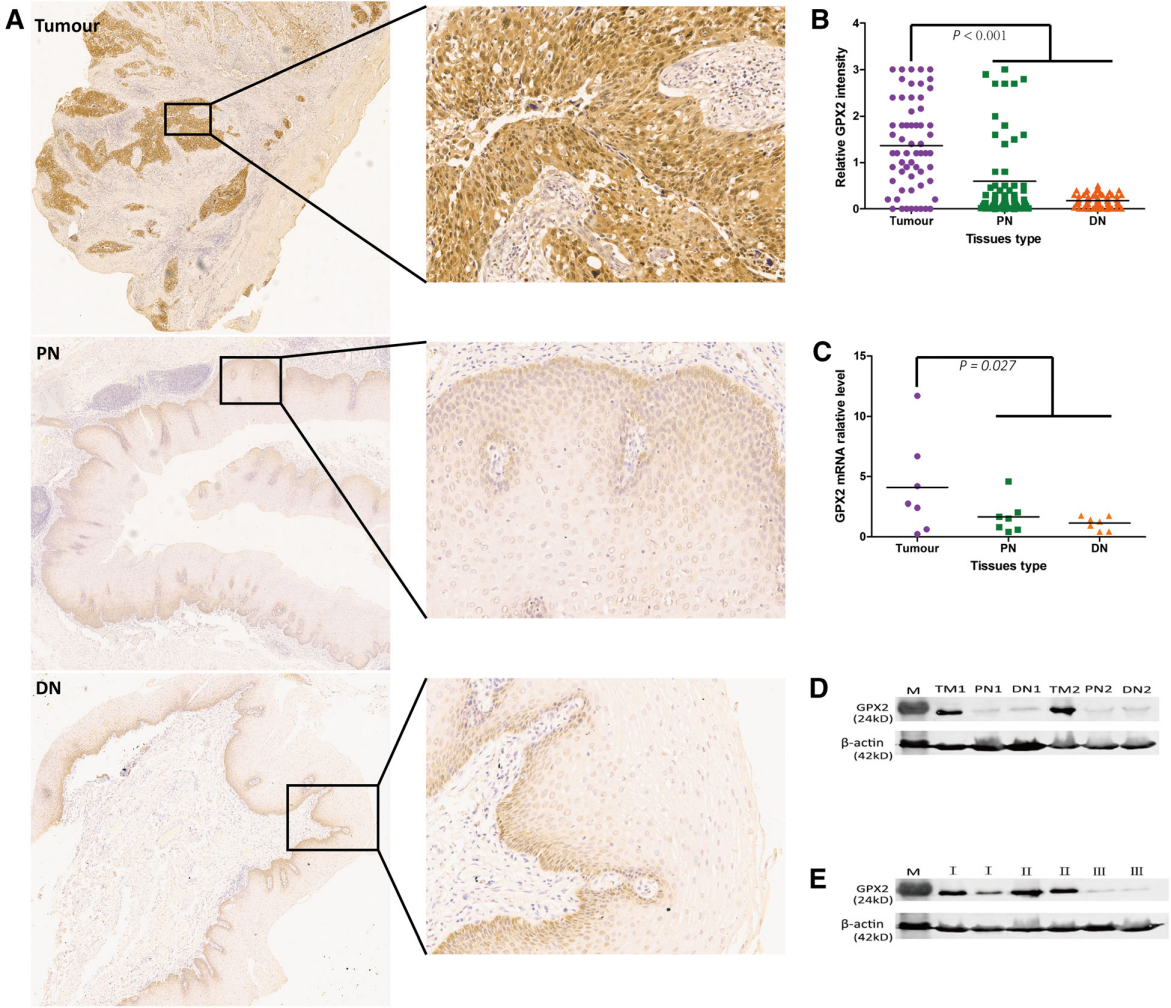
GPX2 overexpression in tumour tissues compared with non-tumour tissues including PN and DN tissues. **a** Representative IHC images of GPX2 staining in tumour, PN and DN tissues. **b** The immunochemistry analysis of the relative expression of GPX2 in tumour, PN and DN tissues according to staining intensity. There is a statistically significant difference in GPX2 protein expression of tumour tissues compared with compared with non-tumour tissues including PN and DN tissues (*P* < 0.001) studied by the independent-samples test. **c** The relative level of GPX2 mRNA expression in tumour, PN and DN tissues. There is statistically significant difference in GPX2 mRNA expression of tumour tissues compared with compared with non-tumour tissues including PN and DN tissues (*P* = 0.027) studied by the independent-samples test. **d** Western blot analysis of GPX2 protein in tumour, PN and DN tissues from two patients within ESCC. Obviously, GPX2 protein was overexpressed in tumor tissues compared with PN and DN tissues. **e** Western blot analysis of GPX2 protein in ESCCI, ESCCII and ESCCIII tissues from six patients with ESCC. Obviously, the expression of GPX2 protein was down-regulated in ESCCIII tissues compared with ESCCI and ESCCII tissues. M: protein marker; TM: tumour;I: ESCCI; II: ESCCII; III: ESCCIII

As detected in other tumours, the expression of GPX2 protein was markedly up-regulated within ESCC tumour tissues. In this study, immunohistochemistry (IHC) results of triple samples of 60 patients with ESCC showed that the significant higher expression level of GPX2 protein was detected in ESCC tumour tissues compared with non-tumour including PN and DN tissues (*P* < 0.001) (Fig. 2a and b). To further verify the IHC results of the expression of GPX2 protein, western blotting and RT-PCR were employed by this study. Under equal-weight protein samples, western blotting results showed that GPX2 was obviously overexpressed within ESCC tumour tissues compared with non-tumour including PN and DN (Fig. 2d and Additional file 2: Figure S2). The interesting phenomenon was also simultaneously demonstrated by the RT-PCR result of GPX2 mRNA (*P* = 0.027) (Fig. 2c). Hence the expression of GPX2 protein was significantly up-regulated within ESCC tumour tissues.

### Expression of GPX2 protein significantly correlated with tumour histological grade of ESCC

According to the IHC results of GPX2 protein expression within ESCC tumour tissues, we divided ESCC patients into two groups, namely GPX2-positive expression (GPX2+) group and GPX2-negative expression (GPX2-) group. We found that GPX2 protein was expressed positively in 82.0 % (132 out of 161) of the cases of ESCC. Further, we analysed the relationship between the expression of GPX2 protein and ESCC patients’ clinicopathological characteristics. We found that the expression of GPX2 protein has statistically significant difference in the tumour histological grade of ESCC (*P* < 0.001), while there were no statistically significant difference in age, sex, tumour size, tumour location, gross morphology and clinical TNM stages (*P* > 0.05) (Table 1). Therefore, the expression of GPX2 protein was significantly related to the tumour histological grade of ESCC. Furthermore, we detected another important phenomenon that GPX2 protein was hardly expressed within ESCCIII compared with ESCCIand ESCCII (Fig. 2e).

**Table 1:** The relationship between GPX2 protein expression and ESCC patients’ clinicopathological characteristics

Clinical features	Cases	GPX2+	GPX2−	*P* value
Age				
≤ 58 years	83	70	13	0.539
> 58 years	78	62	16	
Sex				
Male	117	95	22	0.670
Female	44	37	7	
Size				
≤ 4.5 cm	98	83	15	0.265
> 4.5 cm	63	49	14	
Site				
Upper	13	12	1	0.380
Middle	107	89	18	
Below	41	31	10	
Gross morphology				
Medullary	79	69	10	0.258
Umbrella	11	9	2	
Ulcer	54	40	14	
Stenosis	17	14	3	
Histological grade				
I	46	41	5	<0.001
II	94	86	8	
III	21	5	16	
TNM stages				
I~II	86	75	11	0.118
III	73	55	18	
IV	2	2	0	

GPX2+: ESCC with GPX2-positive expression; GPX2−: ESCC with GPX2-negative expression

Histological grade: I, high differentiated ESCC; II, moderately differentiated ESCC; III, poorly differentiated ESCC

### Negatively-expression of GPX2 protein predicted a poorer prognosis in ESCC patients

**Fig. 3:**
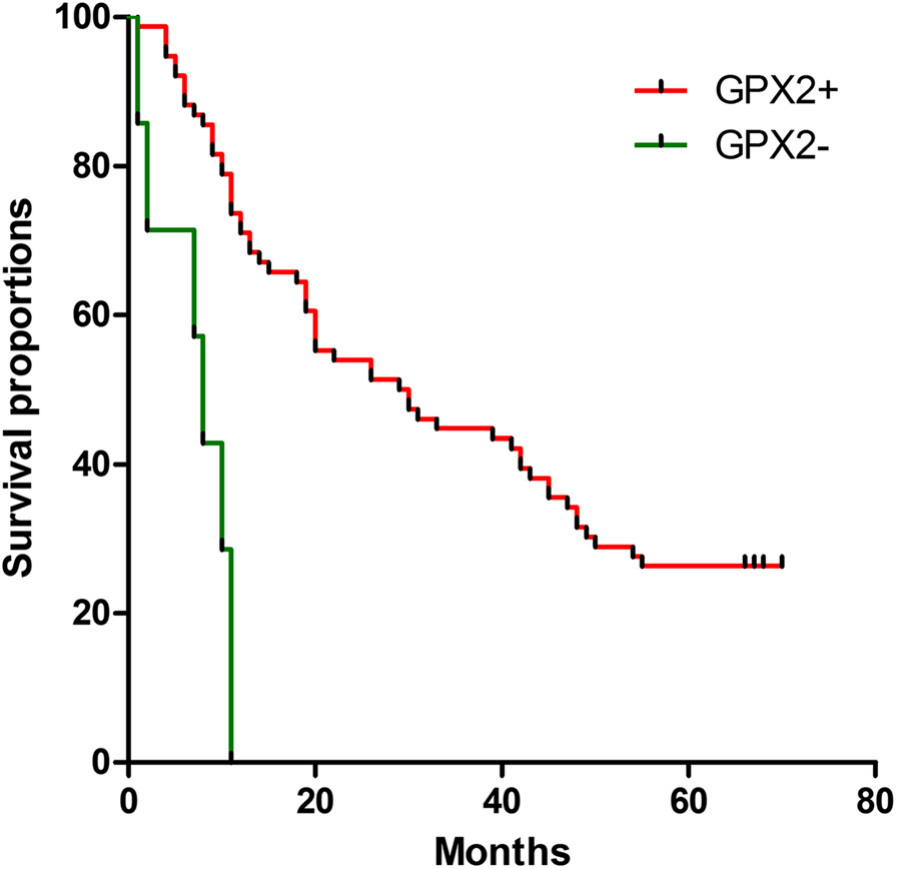
Prognostic analysis of 83 patients with ESCC according to presence of and absence of GPX2 expression. GPX2 positive patients: *n* = 76, GPX2 negative patients: *n* = 7, *P* < 0.001

In the prognostic analysis of 83 ESCC patients with follow-up, firstly, survival analysis of the clinical pathological parameters showed that both tumour histological grade and clinical TNM stages were notably related to the prognosis of patients with ESCC (respectively, *P* = 0.009, HR (95 % CI) = 1.885 (1.212 ~ 2.932); *P* = 0.007, HR (95 % CI) = 2.046 (1.318 ~ 3.177)), while there were no statistically significant differences in age, sex, tumour size, tumour location, gross morphology and radiotherapy or chemotherapy after surgery (*P* > 0.05 or the lower limit of HR (95 % CI) < 1) (Table 2). Secondly, we carried on the prognostic analysis of the expression of GPX2 protein in 83 ESCC patients with follow-up. Those with negative-expression GPX2 in their biopsy specimen had significantly poorer prognosis than those with positive-expression GPX2 (*P* < 0.001) (Table 3 and Fig. 3). One-year survival rates of both positive-expression group and negative-expression group of GPX2 protein were 71.1 % and 0, and median survival time (MST) were 29 (15.117 ~ 42.883) and 8 (5.434 ~ 10.566), respectively (Table 3). In the Cox regression model analysis, we found that the expression of GPX2 protein (*P* < 0.001, HR (95 % CI) = 5.700 (2.337 ~ 13.907)) and tumour histological grade (*P* = 0.01, HR (95 % CI) = 1.739 (1.143 ~ 2.646)) influenced on the long-term survival of ESCC patients, and were death risk factors of ESCC patients (Table 4). Adjusting for the other factor, the death risk of GPX2-negative expression was 4.7 times more than GPX2-positive expression. Thus negatively-expression of GPX2 protein predicted significantly a poorer prognosis in patients with ESCC.

**Table 2:** Survival analysis of the clinical pathological parameters

Clinical features	Cases	Mortality	MST (95 % CI)	Log-rank *P* value	HR (95 % CI)
Age					
≤ 58 years	45	35	26 (16.141~38.859)	0.850	1.049 (0.637~1.725)
> 58 years	38	28	18 (5.918~30.082)		
Sex					
Male	60	46	26 (13.348~38.652)	0.796	1.075 (0.616~1.876)
Female	23	17	18 (8.609~27.391)		
Size					
≤ 4.5 cm	48	35	29 (17.118~40.882)	0.339	1.270 (0.772~2.088)
> 4.5 cm	35	28	19 (4.512~33.488)		
Site					
Upper	9	6	33 (0~73.905)	0.028	1.803 (0.992~3.278)
Middle	65	48	29 (18.467~39.533)		
Below	9	9	11 (5.156~16.844)		
Gross morphology					
Medullary	62	46	30 (9.755~50.245)	<0.001	1.252 (0.942~1.663)
Umbrella	5	4	9 (6.853~11.147)		
Ulcer	13	10	20 (10.605~29.395)		
Stenosis	3	3	6 (4.400~7.600)		
Histological grade					
I	32	20	47 (10.966~83.034)	0.009	1.885 (1.212~2.932)
II	46	38	20 (8.616~31.384)		
III	5	5	8 (5.853~10.147)		
TNM stages					
I~II	49	32	42 (22.796~61.204)	0.007	2.046 (1.318~3.177)
III	32	29	14 (6.608~21.392)		
IV	2	2	-		
Treatment					
No	41	31	19 (3.944~34.056)	0.529	0.855 (0.522~1.402)
Yes	42	32	22 (10.886~33.114)		

Histological grade: I, high differentiated ESCC; II, moderately differentiated ESCC; III, poorly differentiated ESCC

Treatment: Radiotherapy or chemotherapy after surgery

**Table 3:** Survival analysis of 83 patients between GPX2 + and GPX2 −

GPX2	Cases	Death	MST (95 % CI)	One-year survival rate (%)	*χ* ^2^ value	Log-rank *P* value
+	76	56	29 (15.117~42.883)	71.1	22.587	<0.001
−	7	7	8 (5.434~10.566)	0		

GPX2+: ESCC with GPX2-positive expression; GPX2−: ESCC with GPX2-negative expression

**Table 4:** Cox regression of 83 patients with ESCC

Variable	B	SE (B)	Wald *χ* ^2^	*P* value	HR	HR 95 % CI
GPX2	1.741	0.455	14.630	<0.001	5.700	2.337~13.907
Histological grade	0.553	0.214	6.688	0.010	1.739	1.143~2.646

## Discussion

EC is an aggressive malignant neoplasm with a poor prognosis. The 5-year survival rates of patients with EC ranges from 15 to 24% [18]. In China, EC ranks 5^th^ in the most common cancer and 4^th^ in the leading cause of cancer death [19]. The two major histological subtypes of EC is ESCC and esophageal adenocarcinomas (EAC). In contrast to EAC, ESCC has a high prevalence in Asia and South Africa [20]. The consumption of hot food and beverages is considered as an increased risk of esophageal cancer, particularly ESCC [21]. The traditional treatment, like surgery, radiotherapy and chemotherapy, do not bring a greater benefits to patients with EC. Even worse, there is no a better prognostic factor which can predict the life expectancy of patients with EC. Therefore, the prognostic factor of EC should deserve to make it.

Glutathione peroxidases (GPXs) is a family of antioxidant enzymes. The biochemical function of GPXs is to reduce H_2_O_2_ or organic hydroperoxides to water or their corresponding alcohols respectively [22]. At the present, the GPX family consists of eight members: GPX1, GPX2, GPX3, GPX4, GPX5, GPX6, GPX7 and GPX8 [4]. In particular, GPX2, also a selenium-dependent enzyme, is specifically expressed within the gastrointestinal tract in human being [5]. Therefore, GPX2 can be considered as one of the most important defence systems against oxidative damage from the consumption of hot food and beverages. Impressively, GPX2 has an anti-inflammatory and anti-tumour effect in the course of the tumorigenesis [23]. Compared with normal tissues, cancer cells produce a higher level of reactive oxygen species (ROS) [24] and cancer tissues suffer from a severer oxidative damage [25, 26]. As expected, the expression of GPX2 protein is up-regulated within various cancer tissues.

Based on epidemiological study, the reproducibility of a semi-quantitative food frequency questionnaire showed the daily intake of selenium was 81.8~231.2 μg in the diets of Chaoshan inhabitants [27], and in our previous study, the hair selenium level of Chaoshan inhabitants was about 0.48 mg/L [28]. According to the 50~250 μg of suitable range of the daily selenium intake of Chinese residents [29], the selenium supply of inhabitants in Chaoshan region is appropriate. Convincingly, GPX2 protein was significantly overexpressed within ESCC tumour tissues compared with non-tumour tissues in this study. GPX2 may play an important role in anti-tumour within ESCC tumour tissues, while ESCC with a loss expression of GPX2 protein is prone to progress to the poor differentiated ESCC. Because of the limited samples with follow-up, whether or not GPX2 is expected to be a monitoring prognostic factor of patients with ESCC, it is still open for further experimentation.

## Conclusions

To sum up, the expression of GPX2 protein was significantly up-regulated within ESCC tumour tissues compared with non-tumour tissues. GPX2 might be an important predictor for the prognosis of patients with ESCC.

## Abbreviations

ESCC, esophageal squamous cell carcinoma; GPX2, glutathione peroxidase 2

## Additional files

Additional file 1: Figure S1. Representative IHC images of normal rabbit IgG as negative control. Images were captured using a Leica IM50 microscope (Imagic Bildverarbeitung AG, Wetzlar, Germany) at 40× (**A**) and 200× (**B**) magnification. (TIF 4013 kb) Additional file 2: Figure S2. Western blot analysis of GPX2 protein in tumour, PN and DN tissues from other 4 patients with ESCC. Obviously, GPX2 protein was overexpressed in tumor tissues compared with PN and DN tissues. M: protein marker; TM: tumour;I: ESCCI; II: ESCCII; III: ESCCIII. (TIF 1324 kb)
