# Supplementary figures and images for: Clinicopathological and prognostic significance of GPX2 protein expression in esophageal squamous cell carcinoma
Source: BMC Cancer. 2016 Jul 7;16:410. doi: 10.1186/s12885-016-2462-3 (PMC4936229; doi:10.1186/s12885-016-2462-3)

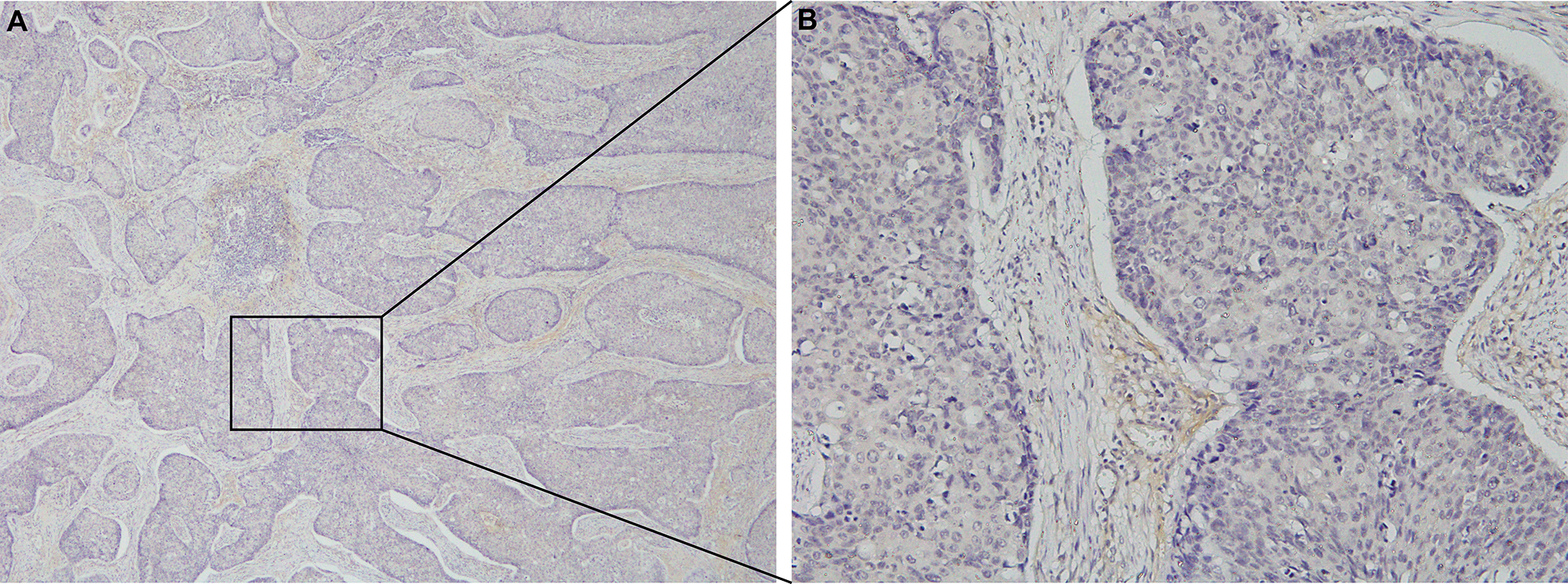

Supplement: Additional file 1: Figure S1. — Representative IHC images of normal rabbit IgG as negative control. Images were captured using a Leica IM50 microscope (Imagic Bildverarbeitung AG, Wetzlar, Germany) at 40× (A) and 200× (B) magnification. (TIF 4013 kb) [file 12885_2016_2462_MOESM1_ESM.tif]
